# Mouse Strain Determines Cardiac Growth Potential

**DOI:** 10.1371/journal.pone.0070512

**Published:** 2013-08-05

**Authors:** Carmen Kiper, Barry Grimes, Gary Van Zant, Jonathan Satin

**Affiliations:** Department of Physiology, University of Kentucky College of Medicine, Lexington, Kentucky, United States of America; National Cancer Institute, United States of America

## Abstract

**Rationale:**

The extent of heart disease varies from person to person, suggesting that genetic background is important in pathology. Genetic background is also important when selecting appropriate mouse models to study heart disease. This study examines heart growth as a function of strain, specifically C57BL/6 and DBA/2 mouse strains.

**Objective:**

In this study, we test the hypothesis that two strains of mice, C57BL/6 and DBA/2, will produce varying degrees of heart growth in both physiological and pathological settings.

**Methods and Results:**

Differences in heart dimensions are detectable by echocardiography at 8 weeks of age. Percentages of cardiac progenitor cells (c-kit+ cells) and mononucleated cells were found to be in a higher percentage in DBA/2 mice, and more tri- and quad-nucleated cells were in C57BL/6 mice. Cardiomyocyte turnover shows no significant changes in mitotic activity, however, there is more apoptotic activity in DBA/2 mice. Cardiomyocyte cell size increased with age, but increased more in DBA/2 mice, although percentages of nucleated cells remained the same in both strains. Two-week isoproterenol stimulation showed an increase in heart growth in DBA/2 mice, both at cardiomyocyte and whole heart level. In isoproterenol-treated DBA/2 mice, there was also a greater expression level of the hypertrophy marker, ANF, compared to C57BL/6 mice.

**Conclusion:**

We conclude that the DBA/2 mouse strain has a more immature cardiac phenotype, which correlates to a cardiac protective response to hypertrophy in both physiological and pathological stimulations.

## Introduction

The heart undergoes structural and morphological changes during maturation and aging [1,2]. Such changes are driven by the requirement of cardiac function to match demand as individuals grow, and age. Natural genetic diversity imposes a broad range of growth curves on human populations. By contrast, inbred mouse strains used in research exhibit distinct aging properties, and therefore provide a potential model system to study growth. A major hindrance is the absence of strain-specific information exploring strain-specific cardiac phenotypes.

While it is unclear how the heart grows and adapts from puberty until senescence, the old model that the heart is a terminally-differentiated organ, whereby cardiomyocytes at birth simply adapt to body growth and aging by changing size, is overwhelmingly unsupported as the sole mechanism [3]. The newer model of self-renewal posits that the heart contains a continuum of pre-myocardial and mature-myocardial cell types ranging from resident cardiac progenitor cells, to immature cardiomyocytes, to mature cardiomyocytes containing increasing nucleation in an age-dependent fashion [4,5]. This paradigm of cardiac turnover has gained more credence in the last couple of years, yet controversial issues persist, particularly in humans where the extremely slow turnover of cardiomyocytes creates experimental barriers [6,7]. Cardiac progenitor cells are stem cells, differentiated to the point whereby they are committed to a cardiac lineage that includes further possible differentiation into smooth muscle cells, endothelial cells, or cardiac myocytes [7–10]. Cardiac progenitor cells are present in the heart in small numbers [11], contain cardiac specific transcription factors [12], and can be isolated by antibodies against c-kit [13,14]. Cardiac progenitor cells differentiate into immature cardiomyocytes [10,15–18] which are characterized as small, mononucleated myocytes and exhibiting similar properties to embryonic-like cardiomyocytes [4,5]. Immature cardiomyocytes can develop into larger, multinucleated mature myocytes – about 80% of mouse heart cardiomyocytes are binucleated [4,5]. The cardiomyocytes with three and four nuclei have the largest area among cardiomyocytes and are considered the oldest cardiomyocytes as these cells have the highest percentage of apoptosis and the least chance of cellular division [4,5]. In this study, we evaluated the response to aging and hypertrophy in two mouse strains.

Genetic background influences physiology in many ways. Inbred mouse strains have been used for years to study specific diseases, but inbred strains often vary with their responses to diseases [19]. Genetic background affects the function of mouse hearts [20], and the response of the heart to experimentally-induced pathological insult [21]. Understanding the contribution of genetic background to physiology under normal conditions is important for selecting appropriate strains as models of disease, and has the potential to provide clues to mechanisms of cardiac growth and repair.

This study is partially motivated by the finding that hematopoietic stem cell (HSC) populations in DBA/2 compared to C57BL6/J mice show distinct properties. HSCs are more abundant in young adult DBA/2 compared to C57BL6/J mice [22] and have a greater proliferative capacity in DBA/2 mice [23]. HSCs from DBA/2 mice show more primitive developmental characteristics than those from C57BL/6 [23]. The raises the provocative hypothesis that differential cardiac progenitor cell population sizes exist in DBA/2 versus C57BL/6 hearts. Our data now support this contention, as DBA/2 hearts contain more c-kit+ cells, and more mononucleated cardiomyocytes in comparison to C57BL/6 hearts.

## Methods

### Animals:

All mice were purchased from Jackson Laboratories and were housed in a pathogen-free facility and treated in accordance with standard use protocols, animal welfare regulations, and the NIH *Guide for the Care and Use of Laboratory Animals*. The animal use protocol was approved by the University of Kentucky Institutional Animal Care and Use Committee.

### Cardiomyocyte Isolation:

Euthanasia was performed by an injection of ketamine+xylazine to anesthetize, followed by cervical dislocation and heart excision. Single ventricular myocytes from female mice were enzymatically isolated following a modified AfCS protocol PP00000125 as previously described. Only rod-shaped, quiescent cardiomyocytes with clear edges were selected for analysis. There was no difference in the percentage of viable rod-shaped cardiomyocytes isolated between the strains. Pictures were taken using a 10x lens. Approximately 200 cardiomyocytes per heart were assessed. Cardiomyocyte area was analyzed through ImageJ software.

### Cardiac Progenitor Cell Isolation:

 The isolation of the cardiac progenitor cells were based on a protocol from Ronglih Liao’s laboratory [24,25]. Mice were anesthetized (ketamine + xylazine) and cervically dislocated. Hearts were excised and placed into cold PBS to rinse out the extra blood. Hearts were individually placed in Petri dishes and minced with 5ml of digestion buffer (2U dispase, 0.1% collagenase, 2mM CaCl_2_, glucose, NaCl and NaHCO_3_^-^) using a razor blade. Solution was mixed with cold HBSS solution buffer (HBSS, 2% FBS (HI), 2mM HEPES), filtered first through a 70µm filter, and then a 40µm filter and stained with 1:100 with antibodies: c-kit (eBioscience), CD45 and CD31 (BD). Solution was filtered a third time with 35µm filter and sorted using a FACS Aria cell sorter. FACS sorts that were outside of the 99% confidence interval were considered outliers. Only cells that were c-kit+/CD31-/CD45- were determined to be cardiac progenitor cells.

### Echocardiography:

Isoflurane combined with oxygen was used to sedate the animals. Heart rate was monitored by ECG and was kept at a constant rate among animals. Echocardiograms were done with the VisualSonic 660 ultrasound machine with the 707-30mHz probe. Images were captured on the short axis parasternal view and measurements were made in M-mode.

### qRT-PCR:

Left ventricular free wall was cut from excised hearts and flash frozen in dry ice and stored at -80^o^C until used. Lysates were made using Trizol reagent. RNA was extracted (Ambion) and cDNA was made (SuperScript II, Invitrogen). Probes were bought commercially from BD. Samples (0.2ng of cDNA) were run with the Taqman PCR Master Mix (Applied Biosystems). Samples were run in triplicate on a 96-well optical plate with the ABI 7700 Sequence Detection Software from Applied Biosystems. All samples were normalized using GAPDH as an internal control.

### Hypertrophic hearts:

Osmotic mini pumps (Alzet, model 2002) were filled with isoproterenol (30mg/kg/day) and were primed overnight. Pumps were inserted subcutaneously into mice. Mice were weighed and echocardiograms were conducted before pump implantation, as well as at two weeks after pump implantation.

### Mitotic Activity:

Mice were given BrdU (0.8mg/ml) in their drinking water for 10 days prior to the experiment, with the water changed daily. At the endpoint, mice were euthanatized, the hearts were perfused with cold PBS and then tissue was fixed with 4% paraformaldehyde. The hearts were then excised and stored in sucrose overnight. They were flash frozen in liquid nitrogen and stored at -80° C until they were ready to be sectioned. Hearts were sectioned at 4µm and then stained with the BrdU visualization kit (Invitrogen). Pictures were taken with a 20x lens on a phase contrast microscope and the BrdU+ nuclei were counted as well as the total area of the section. Areas containing large blood vessels were excluded from analysis. Mouse intestine served as a positive control.

### Apoptotic Activity:

Hearts were prepared for sectioning as described in Mitotic Activity (above). For a positive control, a mouse was injected with doxorubicin IP (100µl, Bedford Laboratories) 48hrs before hearts were taken. Sections were taken at 8µum and frozen (20° C) before used. Sections were stained with the In-Situ Cell Death Detection, POD kit (Roche) and then incubated with fluorescein (PerkinElmer). Positively stained cells were viewed at 20x on a phase-contrast microscope.

### Statistical Analysis:

Experiments were determined significant by the student’s t-test if p < 0.05, or by two-way ANOVA, p < 0.05. All data was analyzed in the software program, PRISM, and are represented as mean ± SEM.

## Results

### Comparison of *in vivo* dimensions and function between strains

Baseline measurements of heart function and size were conducted by echocardiography at 8 weeks of age. Diastolic left ventricular chamber internal diameter was significantly larger in the DBA/2 compared to C57BL/6. C57BL/6 and DBA/2 mice had indistinguishable fractional shortening and ejection fraction. Body weight and heart weight were not different between strains (Table 1). C57BL/6 and DBA/2 mice were therefore considered functionally and structurally similar at 8 weeks of age.

**Table 1:** Basal characteristics of heart function in C57BL/6 and DBA/2 mice.

Parameters		
	DBA/2	C57BL/6
Bw (g)	20.54 ± 0.59	19.26 ± 0.30
IVS d (mm)	0.88 ± 0.06	0.85 ± 0.05
IVS s (mm)	1.09 ± 0.07	1.11 ± 0.06
LVID d (mm)	3.30 ± 0.10	3.05 ± 0.06*
LVID s (mm)	2.28 ± 0.10	2.07 ± 0.07
LVPW d (mm)	0.97 ± 0.05	1.03 ± 0.04
LVPW s (mm)	1.24 ± 0.06	1.29 ± 0.06
FS (%)	31.37 ± 1.95	31.76 ± 1.40
EF (%)	59.19 ± 2.6	60.61 ± 2.01
Hw (mg)	83.04 ± 5.88	78.22 ± 3.67
Hw:Bw (mg/g)	3.89 ± 0.25	4.01 ± .019

All data are represented as mean ± SEM

p < 0.05, student’s t-test

n = 27 (C57BL/6), 24 (DBA/2)

* indicates significant between strains

Abbreviations: BW, body weight; IVS, intraventricular septum; LVID, left ventricular internal diameter; LVPW, left ventricular posterior free wall; EF, ejection fraction; FS, fractional shortening; HW, heart weight; HW:BW, heart weight : body weight; s, systole; d, diastole

### Cardiomyocyte size and degree of nucleation between the two strains

A relatively new paradigm for heart growth homeostasis posits a life-long turnover of cardiomyocytes [12], whereby cardiac progenitor cells differentiate into small, beating cardiomyocytes [8,10]. In turn, mononucleated cardiomyocytes grow in size, become multi-nucleated and develop into fully functioning mature myocytes. In this new paradigm, the fraction of mononucleated myocytes can serve as a crucial indicator of heart growth. We next wanted to assess the relative proportions of mononucleated/binucleated cardiomyocytes in our mouse strains.

**Figure 1:**
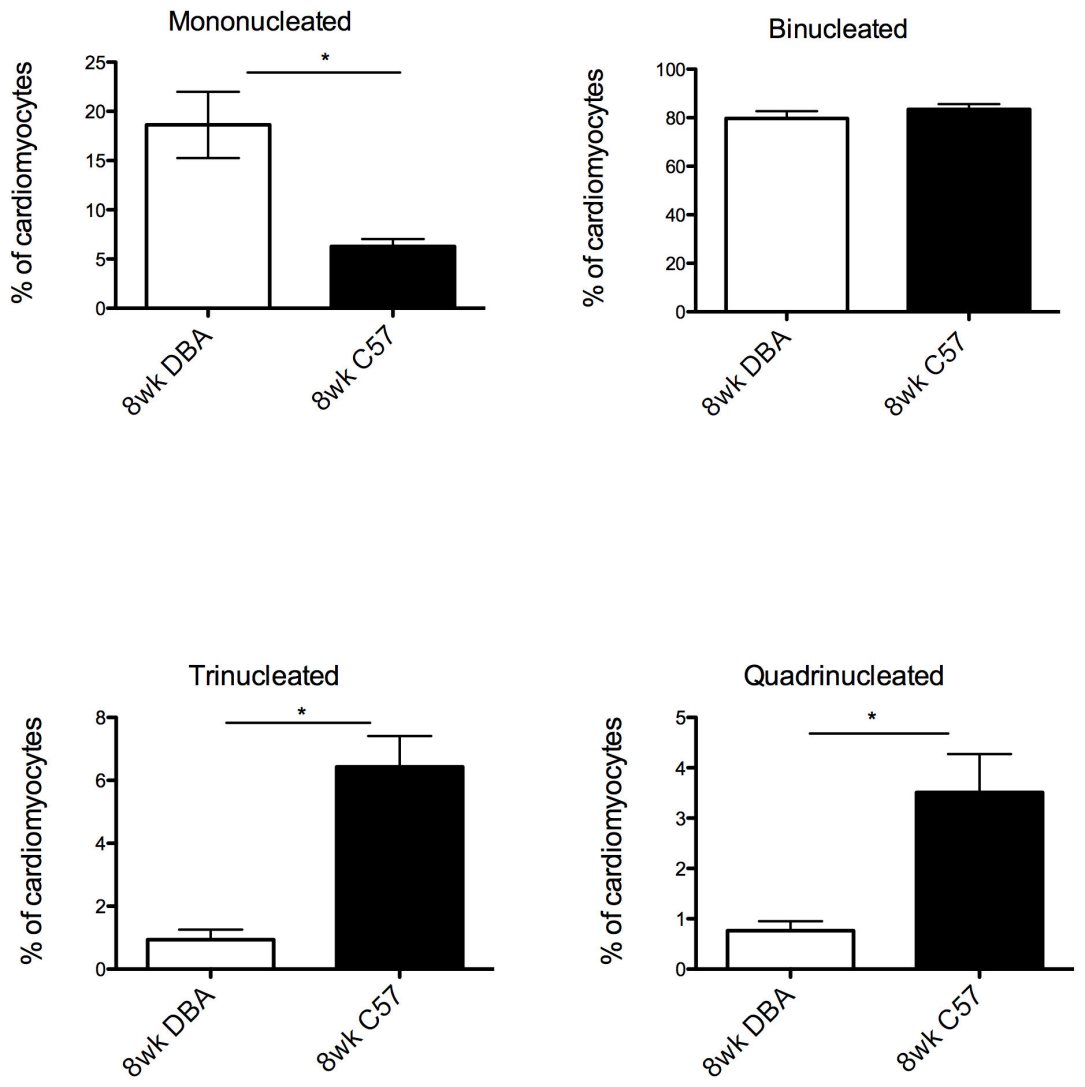
Comparison of cardiomyocyte composition show an immature phenotype for DBA/2 mice. Hearts from seven DBA and eight C57 mice were used for this analysis (200 cardiomyocytes per heart). **A**) Mononucleated cardiomyocyte fraction is significantly higher in DBA than C57Bl/6 hearts; *p<0.05. **B**) Bi-nucleated cells have indistinguishable proportions in both C57BL/6 and DBA/2 mice. **C**) Tri-nucleated cells have a significantly higher percentage in C57BL/6 mice compared to DBA/2. **D**) Quadri-nucleated cells have a significantly higher percentage in C57BL/6 mice compared to DBA/2.

Individual cardiomyocytes were isolated and size and percentage of each number of nuclei were measured. The fraction of mononucleated cardiomyocytes (relative to total cardiomyocytes) is significantly greater in DBA/2 compared to C57BL/6 mice. DBA/2 mice also had fewer tri- and quadri-nucleated cells compared to C57BL/6 (Figure 1). Cardiomyocyte size, for any given degree of nucleation, was not significantly different between the two strains (data not shown). Thus, based on an increased fraction of mononucleated cardiomyocytes and a commensurate decreased fraction of tri- and quadri-nucleated cardiomyocytes, we conclude that the 8 week DBA/2 hearts have a relatively immature phenotype compared to C57BL/6 heart.

### Karyokinetic and apoptotic activity between the two strains

In order to increase the number of nuclei within the cell, the nucleus must replicate without cell division - termed karyokinesis. We next measured the karyokinetic activity in C57BL/6 and DBA/2 mice to determine if this could account for the different percentages of nuclei. We focused on the apex of the heart because of the reported concentration of cardiac progenitor cells [10]. C57BL/6 mice had more mitotically active cells, although the difference was not statistically significant (Figure S1 A,C). Apoptosis was also measured in C57BL/6 and DBA/2 hearts to further measure the turnover ability of the cardiomyocytes. As cardiomyocytes get older, larger and produce more nuclei, they will also increase their chance of undergoing apoptosis [4,26]. TUNEL staining was used to stain for apoptotic cells in histological sections of the apex of both strains. C57BL/6 mice had a greater number of positively stained cells compared to DBA/2, although the difference did not reach statistical significance (Figure S1B,D).

### Cardiomyocyte size with aging

**Figure 2:**
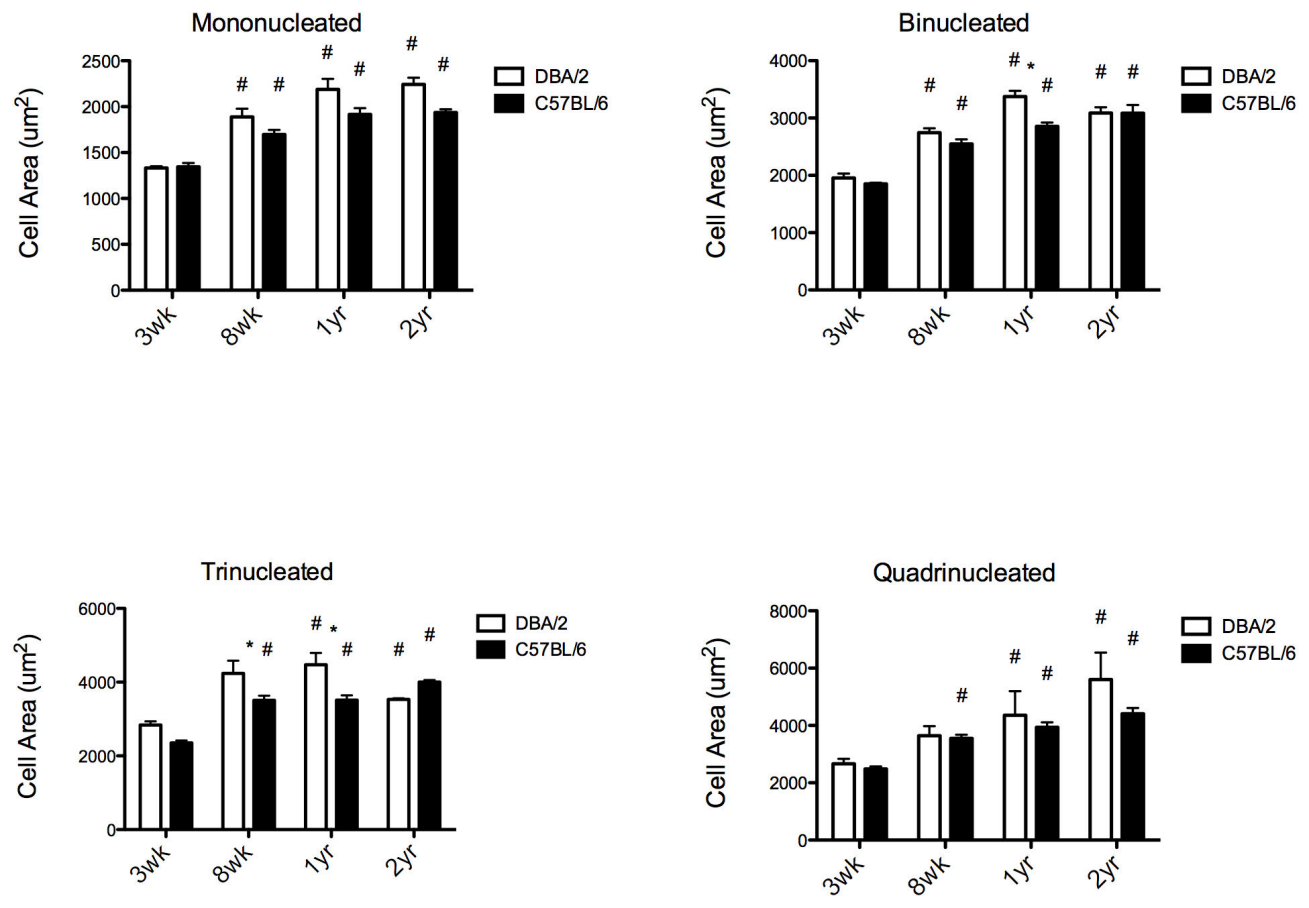
Average cardiomyocyte size increases with age for DBA/2 and C57BL/6 hearts; greater increase for DBA/2. Individual cardiomyocytes were isolated from hearts (n = 3, 7, 3 for 3-week, 8-week, and 1-year, respectively) from DBA/2, and (n = 4, 8, 4) from C57BL/6 mice. Approximately 200 cardiomyocytes were counted from each heart. **A**) Mononucleated cardiomyocytes are significantly larger in both strains with aging. **B**) Binucleated cardiomyocytes are significantly larger with aging for both strains, and DBA/2 cardiomyocytes are also significantly larger than C57BL/6 by 1 year of age. **C**) Tri-nucleated cardiomyocytes increase in size between 3 weeks and 8 weeks of age for both strains, and DBA/2 mice have significantly larger cardiomyocytes compared to C57BL/6 by 8 weeks as well as at 1 year. **D**) Quadri-nucleated cardiomyocytes are significantly larger from 3 weeks in the C57BL/6 mice at 8wks and at 1 year of age for both strains. #: indicates significant from 3 weeks of age, *: indicates significant between strains; p < 0.05, 2-way ANOVA.

**Figure 3:**
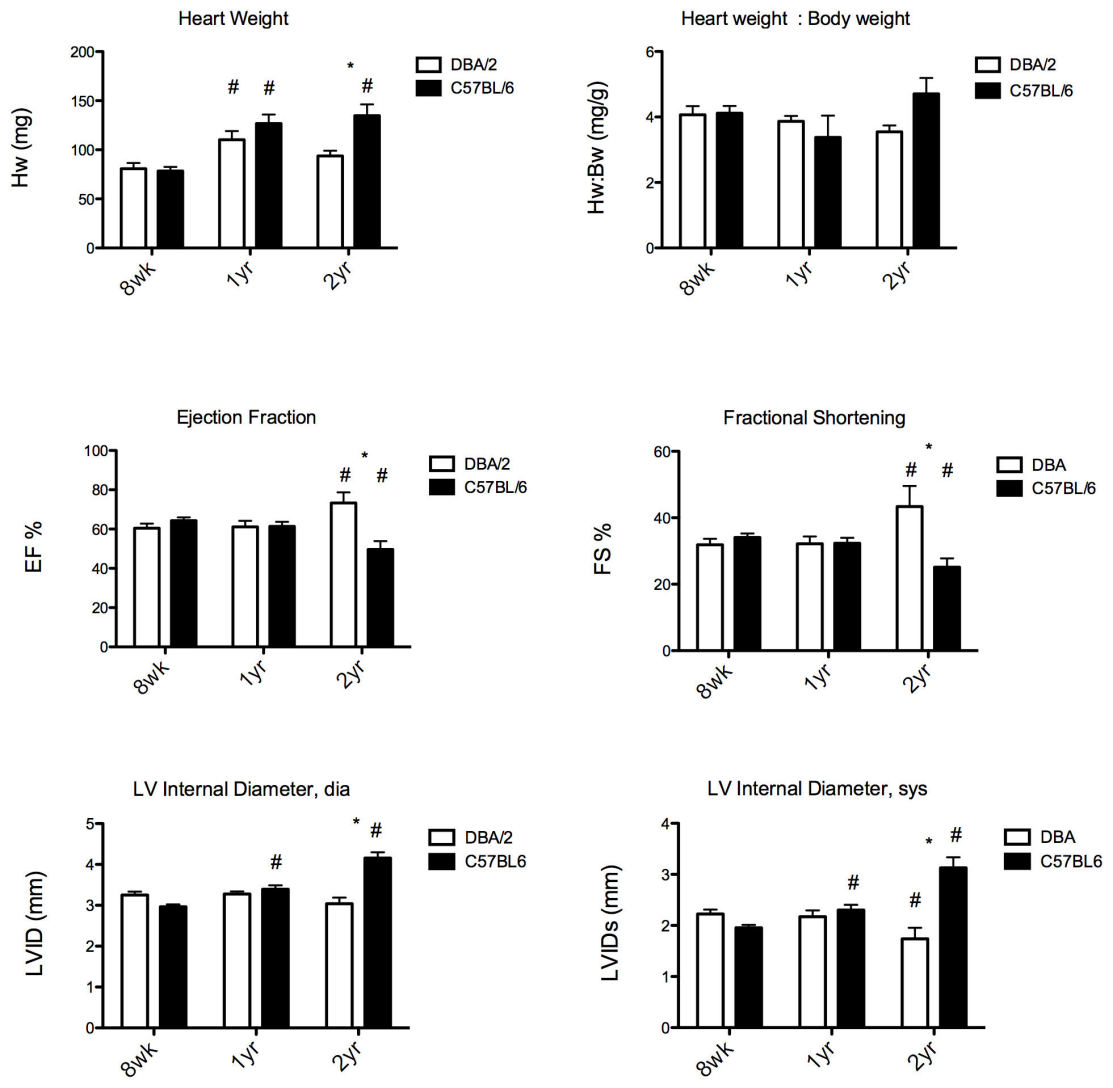
Old C57BL/6 mice show a decrease in function with aging. **A)** Heart weight increases in both strains with aging, C57BL/6 mice increase the most. **B)** Heart weight : body weight tends to increase with aging, with C57BL/6 increasing the most, although not significantly. Cardiac function as shown through ejection fraction **(C)** and fractional shortening **(D)** is decreased in C57BL/6 mice with aging, whereas it is preserved in DBA/2 mice. Left ventricular internal diameter in diastole **(E)** and systole **(F)** show a dilated chamber in C57BL/6 mice with aging and a preserved chamber diameter in DBA/2 mice with aging. *: indicates significant between strains, #: indicates significant from 8 weeks of age; p < 0.05, 2-way ANOVA.

Previous studies showed that the number of mononucleated and multinucleated cardiomyocytes were dependent on age of the animal, with younger animals containing more mononucleated, or immature, cardiomyocytes [4,5]. We next tested the hypothesis that there were differences in percentage of nuclei with these two strains in both young and old animals. Cardiomyocytes were isolated from mice at 3 weeks, 8 weeks, 1 year, and 2 years of age. Contrary to previous results [4,5], the difference in the percentage of nucleated cardiomyocytes across ages for within each strain was not strikingly different (Figure S2 A–D). However, the cardiomyocyte cell area increased with age for all numbers of nuclei. The increase in cell size was greatest in the DBA/2 mice (Figure 2). Overall heart size was measured in 1 year and 2 year old mice and compared to 8 week old mice (3 week mice were too small to conduct echocardiograms). Heart size increased in both strains, but increased in C57BL/6 mice the most (Figure 3A). Heart function, as shown through ejection fraction and fractional shortening, was preserved in DBA/2 mice with aging, however C57BL/6 mice had a decrease in cardiac function, resembling heart failure (Figure 3C,D). Individual measurements of the left ventricular internal diameter show a dilated ventricle with aging in C57BL/6 mice, again indicating a failing heart, whereas DBA/2 mice showed no change in their ventricular diameter with aging (Figure 3 E,F)*.*

### Cardiac progenitor cells in C57BL/6 and DBA/2 mice

**Figure 4:**
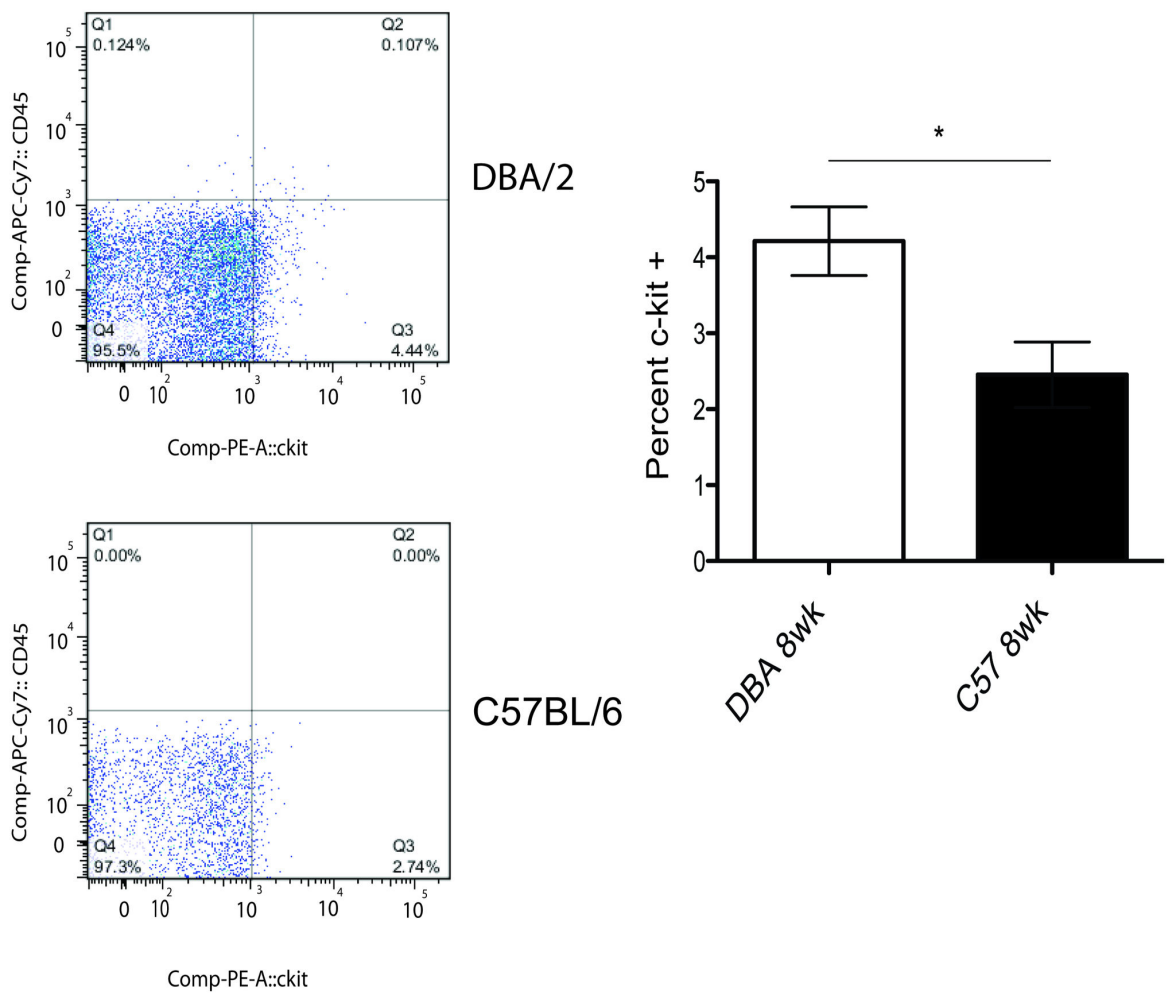
DBA/2 mice have a greater percentage of c-kit+ cardiac progenitor cells. C-kit+ cells were isolated through flow cytometry. Only cells that were negative for CD31 and CD45 were considered cardiac progenitor cells. **Left**) Representative flow cytometry showing c-kit+/CD45-/CD31- progenitor cells in C57BL/6 and DBA/2 mice (third quadrant, Q3). **Right**) DBA/2 mice have an average of 4.2% of c-kit+ progenitor cells compared to C57BL/6 mice, which have an average of 2.5% progenitor cells; n = 7,8; *p < 0.05, student’s t-test.

DBA/2 mice had a greater number of primitive hematopoietic stem cells at a young age than C57BL/6 mice [22]. This motivated the hypothesis that there would also be similar strain-specific differences in the number of progenitor cells found in the heart. Furthermore, more progenitor cells in the heart would indicate a less differentiated, more immature heart. To determine the number of progenitor cells, we assayed hearts for the common cardiac progenitor marker, c-kit [10,13,14,27]. Cells that were also positive for CD45 (hematopoietic) and CD31 (endothelial) were excluded [25,28]. DBA/2 mice had significantly greater percentages of progenitor cells compared to C57BL/6 mice at 8 weeks of age (Figure 4). C-kit+ cells were also isolated at 2 years of age in both strains. By this age, C57BL/6 and DBA/2 mice had similar percentages of c-kit+ cells (*data not shown*)*.*

**Figure 5:**
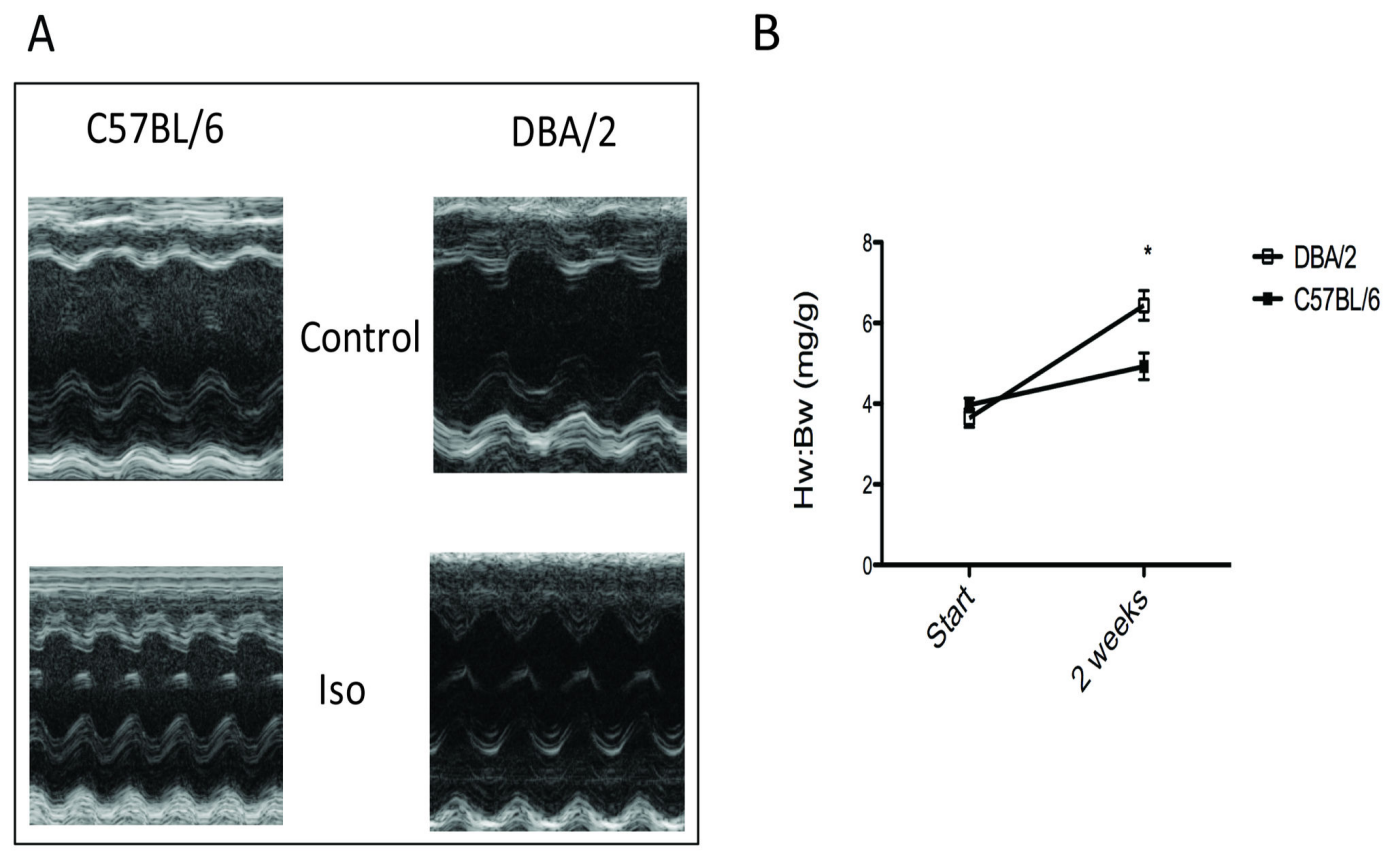
Response to isoproterenol treatment is greater in DBA/2 mice compared to C57BL/6. **A**) Representative figures of echocardiograms of the left ventricle before and after isoproterenol treatment. **B**) Heart weight/Body weight ratio shows a greater increase in DBA/2 hearts in response to isoproterenol treatment compared to C57BL/6 mice; n = 9, 10; p < 0.05, 2-way ANOVA.

**Table 2:** Heart function in C57BL/6 and DBA/2 mice following two weeks of isoproterenol treatment.

Parameters	Treatment			
	C57BL/6 Control	C57BL/6 Isoproterenol	DBA/2 Control	DBA/2 Isoproterenol
Bw (g)	20.04 ± 0.33	21.75 ± 0.31	22.03 ± 0.63	21.93 ± 0.56
IVS d (mm)	0.7373 ± 0.038	0.9164 ± 0.066 #	0.8458 ± 0.043	0.9633 ± 0.04
IVS s (mm)	0.9609 ± 0.046	1.463 ± 0.091 #	1.078± 0.052	1.356 ± 0.056 #
LVID d (mm)	3.194 ± 0.079	2.997 ± 0.107	3.397 ± 0.115	3.541 ± 0.083*
LVID s (mm)	2.289 ± 0.061	1.573 ± 0.119 #	2.389 ± 0.073	2.343 ± 0.095*
LVPW d (mm)	0.9727 ± 0.061	1.44 ± 0.11 #	1.001 ± 0.062	1.439 ± 0.077 #
LVPW s (mm)	1.193 ± 0.065	1.79 ± 0.081 #	1.277 ± 0.062	1.743 ± 0.067* #
FS %	28.25 ± 1.22	47.27 ± 3.72 #	29.41 ± 1.26	33.99 ± 1.66* #
EF %	55.74 ± 1.80	78.21 ± 3.84 #	57.27 ± 1.78	63.5 ± 2.33*
Hw (mg)	71.88 ± 3.35	106 ± 6.86 #	90.35 ± 7.33	140.3 ± 7.87* #
Hw:Bw (mg/g)	3.583 ± 0.15	4.924 ± 0.331 #	3.961 ± 0.30	6.435 ± 037* #

All data are expressed as mean ± SEM

p < 0.05, student’s t-test

n = 11 (C57BL/6), 12 (DBA/2)

# indicates significant from control

* indicates significant between strains

Abbreviations: BW, body weight; IVS, intraventricular septum; LVID, left ventricular internal diameter; LWPV, left ventricular posterior free wall; EF, ejection fraction; FS, fractional shortening; Hw, heart weight; Hw:Bw, heart weight : body weight; s, systole; d, diastole

**Figure 6:**
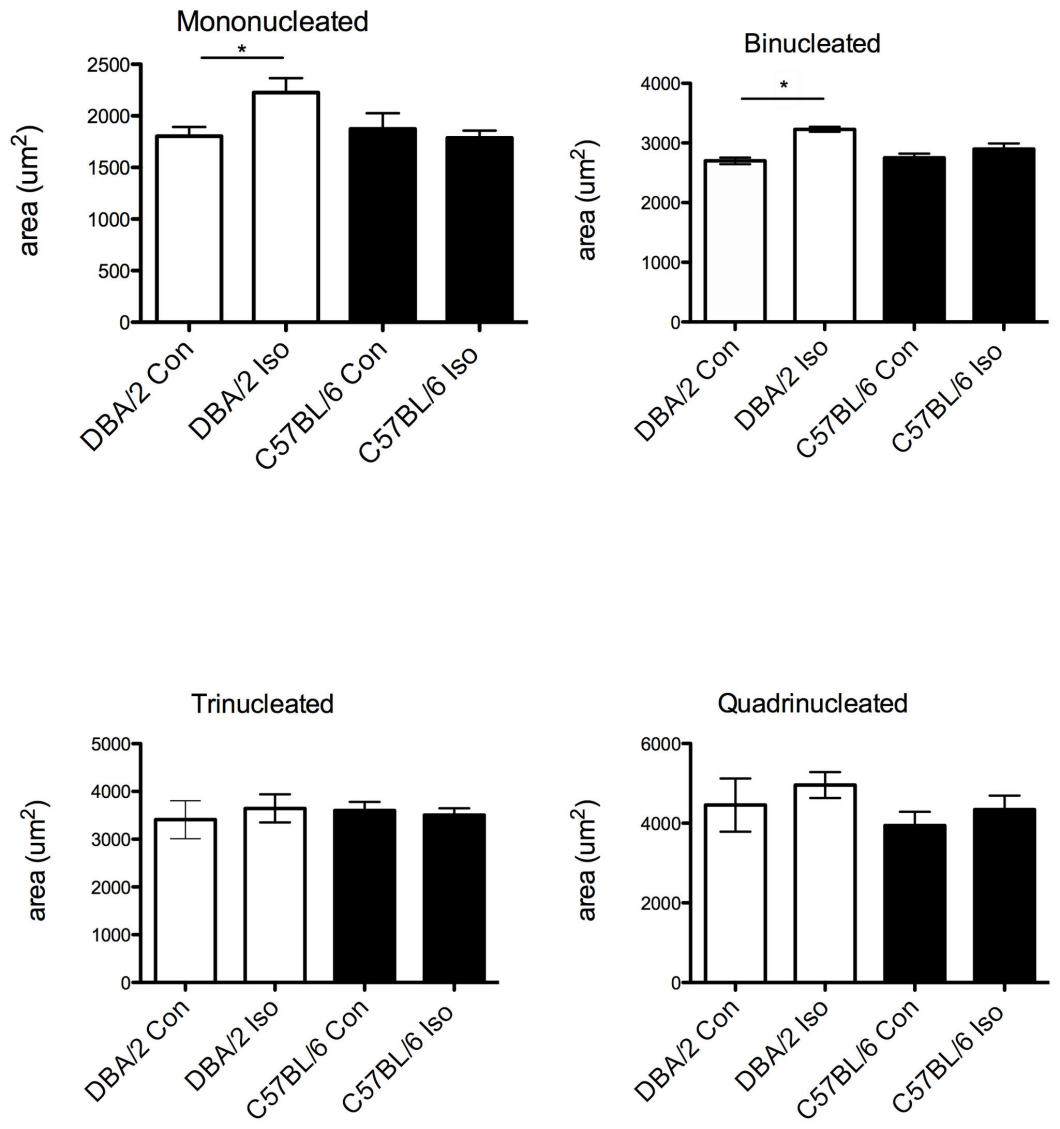
Cardiomyocyte area is increased in isoproterenol-treated DBA/2 mice, but not in isoproterenol-treated C57BL/6 mice. Individual cardiomyocytes were isolated from (n = 4) DBA/2 and (n = 5) C57BL/6 hearts. Approximately 200 cardiomyocytes from each strain were counted. **A**) Mononucleated and (**B**) Binucleated cardiomyocytes show an increase in cell size with isoproterenol treatment in DBA/2 mice. **C**) Tri-nucleated and (**D**) Quadri-nucleated cardiomyocytes show no significant difference in size with isoproterenol treatment in either strain; *p< 0.05.

**Figure 7:**
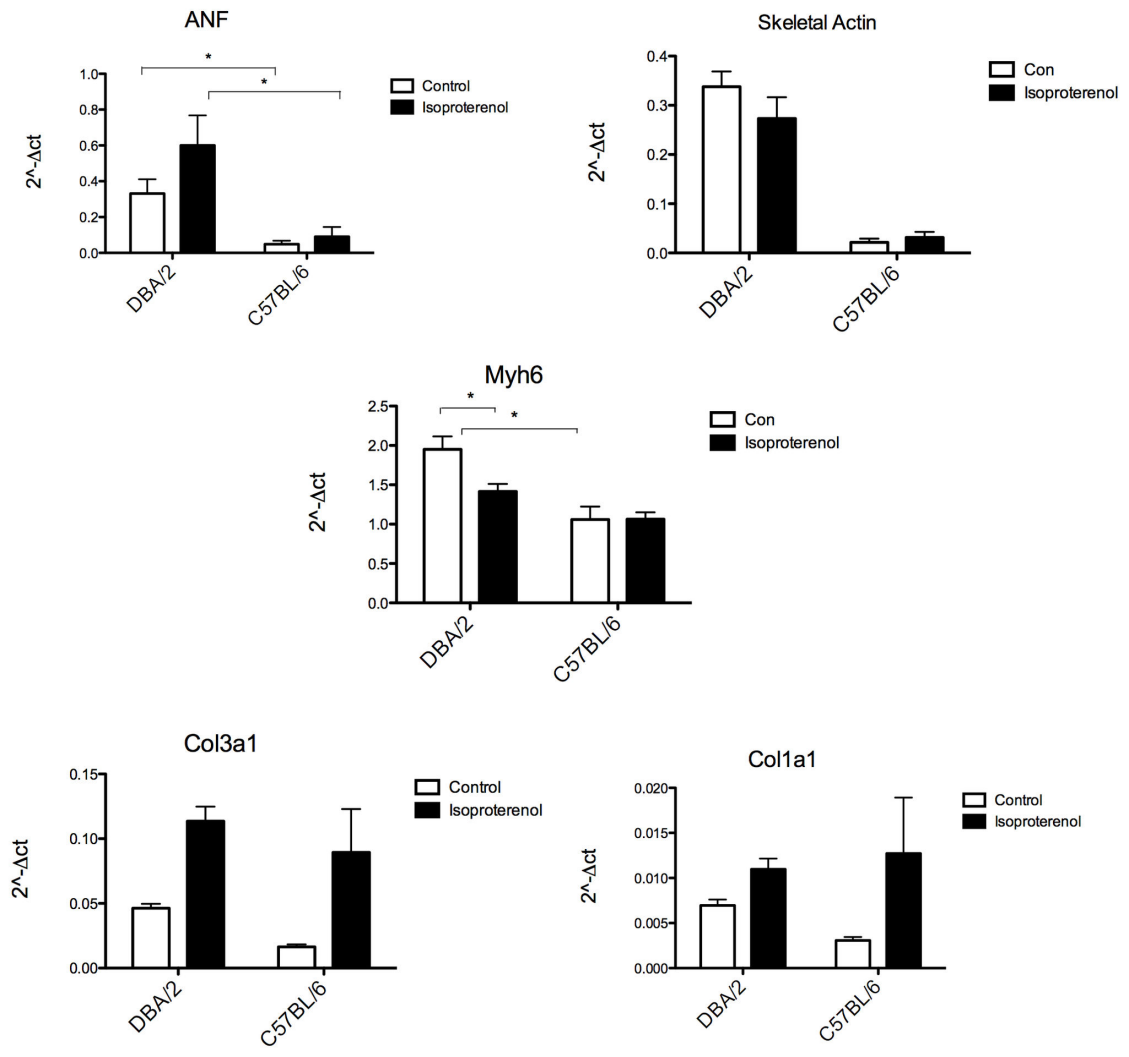
mRNA levels of hypertrophic markers for DBA/2 have greater increase with isoproterenol compared to C57BL/6. A) ANF mRNA level shown in reference to GAPDH mRNA, B) BNP mRNA levels, C) skeletal actin mRNA levels, D) αMHC mRNA levels, E) procollagen type3 a1, F) procollagen type 1a1; n = 3 (for control DBA/2) and 4 for all others; *p < 0.05.

### Isoproterenol stimulation shows greater response in DBA/2 mice.

Isoproterenol is a well-established model for concentric hypertrophy [29] and prolonged infusion eventually leads to heart failure [30]. However, C57BL/6 mice are relatively non-responsive compared to other strains for shorter isoproterenol infusion periods [31,32]. Given that DBA/2 mice exhibit a relatively immature heart compared to the C57BL/6, we tested the hypothesis that DBA/2 mice had a greater heart growth response range than C57BL/6 mice. In another cohort of animals, heart size and function were measured via echocardiography before and after continual isoproterenol infusion to assess change in heart size and showed no difference at baseline. DBA/2 mice showed a greater increase in heart weight to body weight ratio compared to C57BL/6 mice (Figure 5, Table 2). Individual cardiomyocytes were isolated, and cell area measured. Similarly, isoproterenol-treated DBA/2 mice had an increase in cardiomyocyte cell area for mononucleated and binucleated cardiomyocytes compared to control (Figure 6). Relatively large tri- and quadri-nucleated cardiomyocytes did not significantly increase in area. Isoproterenol-treated C57BL/6 mice were not significantly increased (Figure 6). Percentages of nuclei were analyzed and were not significantly changed with isoproterenol-treatment in either strain, similar to what has been shown in the literature [33] (data not shown). Next, mRNA levels of atrial natriuretic factor (ANF) were measured as an independent marker of hypertrophic response to isoproterenol. Consistent with differential growth measures, DBA/2 mice treated with isoproterenol had a larger increase in ANF expression levels in comparison to the increase from isoproterenol-treated C57BL/6 mice (Figure 7A). ANF is also found in more embryonic tissues, and its expression is recapitulated as a fetal response gene. Since baseline levels of ANF were elevated in DBA/2 mice, several other mRNA levels of fetal response genes were measured to determine if DBA/2 mice displayed high levels of other fetal genes. Similar to ANF, message levels of skeletal actin were elevated in DBA/2 mice, whereas the adult form of MHC (αMHC, also myh6) was not up-regulated in either mouse strain (Figure 7B,C). Isoproterenol-induced hypertrophy can result in an increase of extracellular matrix deposition [34]. Message levels of several types of collagen were measured between the two strains. C57BL/6 showed more collagen with isoproterenol compared to isoproterenol-treated DBA/2 mice (Figure 7D,E).

## Discussion

The major new finding in this study was that cardiac hypertrophy, both age-related and pathological-stress-induced, occurs in a strain-specific manner. This is the first study to show that DBA/2 and C57BL/6 mice vary in the composition of cardiomyocytes that are contained in the heart; DBA/2 mice have more cardiac progenitor cells, more mononucleated cardiomyocytes and C57BL/6 mice have more tri- and quadri-nucleated cardiomyocytes. DBA/2 mice also display fewer cells undergoing cell cycling, manifested as fewer mitotic and apoptotic cells. DBA/2 mice also have larger cardiomyocytes at every age, albeit not significantly, and have a greater cardiac hypertrophic response to isoproterenol treatment than C57BL/6 mice.

This study draws on the paradigm that the mature heart, at any given age, contains an admixture of cardiomyocyte precursors, and cardiomyocytes of varying ages [35]. A relatively immature heart would then be expected to contain a larger fraction of mononucleated cells, whereas maturing hearts steadily increase multi-nucleation of cardiomyocytes [4,5]. C-kit+ cells increase with aging and disease [17], and our results now show an aging-dependent relative change in c-kit+ cells between C57Bl/6 and DBA strains. An interpretation of this result suggests that in C57Bl/6 c-kit+ cell growth is more plastic. In this context, our results demonstrating more c-kit+ cells and relatively more mononucleated cells in DBA/2 versus C57BL/6 hearts suggests that the genetic background of DBA/2 mice causes an immature heart phenotype at 8 weeks of age.

Unexpectedly, our data show discordance between the degree of multi-nucleation and cell size as a function of aging. We observed an increase of cardiomyocyte size, but not an increase in the fraction of multi-nucleated cardiomyocytes from 8 weeks to 2 years. In contrast, recent studies indicated that there were differences in percentages of nuclei with age [4,5], specifically that there were more mononucleated cells in younger animals and more multinucleated cells in older animals. Similar to the present study, human heart aging, cardiac hypertrophy, and ischemic cardiomyopathy do not affect the proportion of mono- and multinucleated cardiomyocytes [33]. It is unclear why these studies show discrepant results. The two studies showing age-dependent increase of proportion of multi-nucleated cardiomyocytes were done in cat and mouse (strain C57BL/6 [4,5],, whereas the present study in C57BL/6 mice and the human study show no age dependent change in proportions of multi-nucleation. Our finding that both strains maintain the same percentage of cells indicates that nucleation status is not necessarily part of normal aging. The consistent finding that cardiomyocytes undergo hypertrophy, for any given nucleation number, and as a function of age, suggests that signaling pathways, distinct from karyokinesis, influence normal aging-related heart growth.

The finding that there is a static proportion of mono- to multi-nucleated cardiomyocytes as a function of age does not necessarily preclude an influence of karyokinesis for cardiac growth homeostasis. Previous studies showed that myocyte maturation is commensurate with increasing cell size as well as increasing the number of nuclei [4,5]. Cardiomyocytes and cardiac progenitor cells can also divide to replenish the reserve pool from the cells that have differentiated [14,36]. Our hypothesis was that the C57BL/6 hearts have more multi-nucleated cells, and therefore would have an increase in mitotic activity to create that many nuclei. We focused on the apex of the heart for these measurements because this region is enriched with CPCs [10], and thus allowed for greater resolution in measurements. There was a trend for the increased mean BrdU+ cells in C5BL/6 compared to DBA/2. Moreover, it is interesting to note that the relationship between mitotic activities in C57BL/6 versus DBA/2 mice is correlated in the apoptotic activity between strains. These data suggest the speculative hypothesis that C57BL/6 undergo more cardiomyocyte turnover in the young adult heart. Turnover rate has been shown to be changed under certain condition, such as that associated with an increase in mitotic activity under pathological conditions [37,38] or an increase in apoptotic activity in aging [39,40]. A limitation of this study is that all dividing cells and apoptotic cells were measured, and no specific measurements between dividing cardiomyocytes versus dividing CPCs versus dividing fibroblasts were made. The finding that C57BL/6 have a greater fractional collagen gene expression response to isoproterenol is consistent with the notion that there might be differential fibroblast populations across strains.

C-kit+ cell populations are strain specific, and the increased number of c-kit+ cells in DBA/2 versus C57BL/6 correlates with similar relationships in HSC pools [23]. The present study only considers cells negative for CD31 and CD45 to preclude endothelial and bone marrow cell contributions to our measurements. Although we document relatively more c-kit+ cells in DBA/2 hearts, the contribution of cardiac resident c-kit+ cells is controversial. C-kit+ cells isolated from neonatal hearts can differentiate into cardiomyocytes, but the cardiomyogenic potential of adult c-kit+ cells is not apparent in cells from normal adult heart [41]. Cardiac progenitor cells can contribute to the growth potential of the heart through their differentiation into cardiomyocytes in many different conditions, including the addition of growth factors [18,42,43], as well as after an infarction [44]. There has also been some evidence that progenitor cells will proliferate under hypertrophic conditions [17]. These findings in concert with our present study raise the speculative, but exciting notion that for certain pathologies, and in certain genetic backgrounds, the possibility exists for variable degrees of intrinsic cardiac repair capabilities. Understanding the signaling mechanisms behind progenitor cell proliferation/differentiation will important to be able to utilize progenitor cells as a therapy for myocardial infarctions and other forms of heart disease.

There are many models of cardiac hypertrophy, but we focused on isoproterenol-induced hypertrophy because of the well-documented diminished response that C57BL/6 mice have to isoproterenol stimulation [31]. Thus the differential growth response between the two strains would be more discernable. C57BL/6 mice have been shown to have a decreased response to isoproterenol from their down-regulation of β-adrenergic receptors [31,32], which might contribute to the small increase in heart weight to body weight in the C57BL/6 mice compared to DBA/2. However, there is also the possibility that the DBA/2 mice have a greater growth potential, as evidenced by the large increase in heart weight to body weight with isoproterenol. Also, the mono- and binucleated cardiomyocytes in DBA/2 mice were responsive to isoproterenol treatment, which comprise the majority of cardiomyocytes within the DBA/2 heart. This illustrates the idea that the relatively immature cardiomyocytes are the ones that have the greater growth potential. Equally compelling are the message levels of ANF in baseline, which is typically found in the embryonic heart, and is also part of the recapitulated fetal response genes seen in the hypertrophic and failing heart [45,46]. This again supports the idea that the DBA/2 mouse strain has an immature cardiac phenotype. ANF is typically released during high blood pressure, which increases diuresis to reduce volume, thereby reducing blood pressure. High baseline levels of ANF in DBA/2 mice could be due to higher resting blood pressure. However, other groups describe similar blood pressure levels between DBA/2 and C57BL/6 mice [47], so this is not a likely explanation. Similar to aging, we did not see any alteration in the proportion of nucleated cells with isoproterenol treatment, which again has been shown in human studies [33], but contrary to those done in rats [48]. A possible explanation for these discrepancies could be from the single time-points in this study, giving a ‘snapshot’ view of the change in proportions of nucleated cardiomyocytes. The present study highlights that the mono- and bi-nucleated cardiomyocytes appear to be the major contributory factor to the strain-specific cardiac growth observed with isoproterenol treatment. The greatest degree of growth occurs in the mononucleated cardiomyocytes, which are significantly more abundant in the DBA/2 mice compared to C57BL/6 mice.

We conclude that the DBA/2 and C57BL/6 mouse strains may cycle their cardiomyocytes at different rates, but retain the specific proportions of degree of nucleation of cardiomyocytes during the period of growth from 3 weeks to 2 years. The DBA/2 mouse contains more mononucleated myocytes, and displays a greater range of responsiveness to hypertrophic stimuli as well as preserved function with aging. In summary, this work indicates that genetic background influences the composition of cardiomyocytes within the heart, and that this specific composition has the potential to limit (or aid) response to pathological insults.

## Supporting Information

Figure S1 Mitotic and apoptotic activities tend to be greater in C57BL/6 compared to DBA/2 mice. **A)** Representative picture of mitotic activity manifested as BrdU staining. Scale bar represents 100µm. **B)** Representative picture of apoptotic activity assessed by TUNEL staining. Below: Dapi staining of same slices. Scale bar = 100µm. **C)** C57BL/6 mice have a higher although not significant number of mitotically active cells in the heart compared to DBA/2 mice, n = 4. **D)** C57BL/6 mice have a higher number of apoptotically active cells in the heart compared to DBA/2 mice, however not statistically significant, n = 5.
(TIF)

Figure S2 Percentage of cardiomyocytes remains constant with aging within each strain. **A**) Mononucleated cardiomyocyte percentages remain higher in DBA/2 mice from age 3wks to 2yrs. **B**) Binucleated cardiomyocyte percentages are similar in both strains from age 3wks to 2yrs. And tri-nucleated **C**) and quadri-nucleated **D**) cardiomyocytes percentages are higher in C57BL/6 mice from age 3wks to 2yrs.
(TIF)
