# Supplementary figures and images for: Mouse Strain Determines Cardiac Growth Potential
Source: PLoS One. 2013 Aug 5;8(8):e70512. doi: 10.1371/journal.pone.0070512 (PMC3734269; doi:10.1371/journal.pone.0070512)

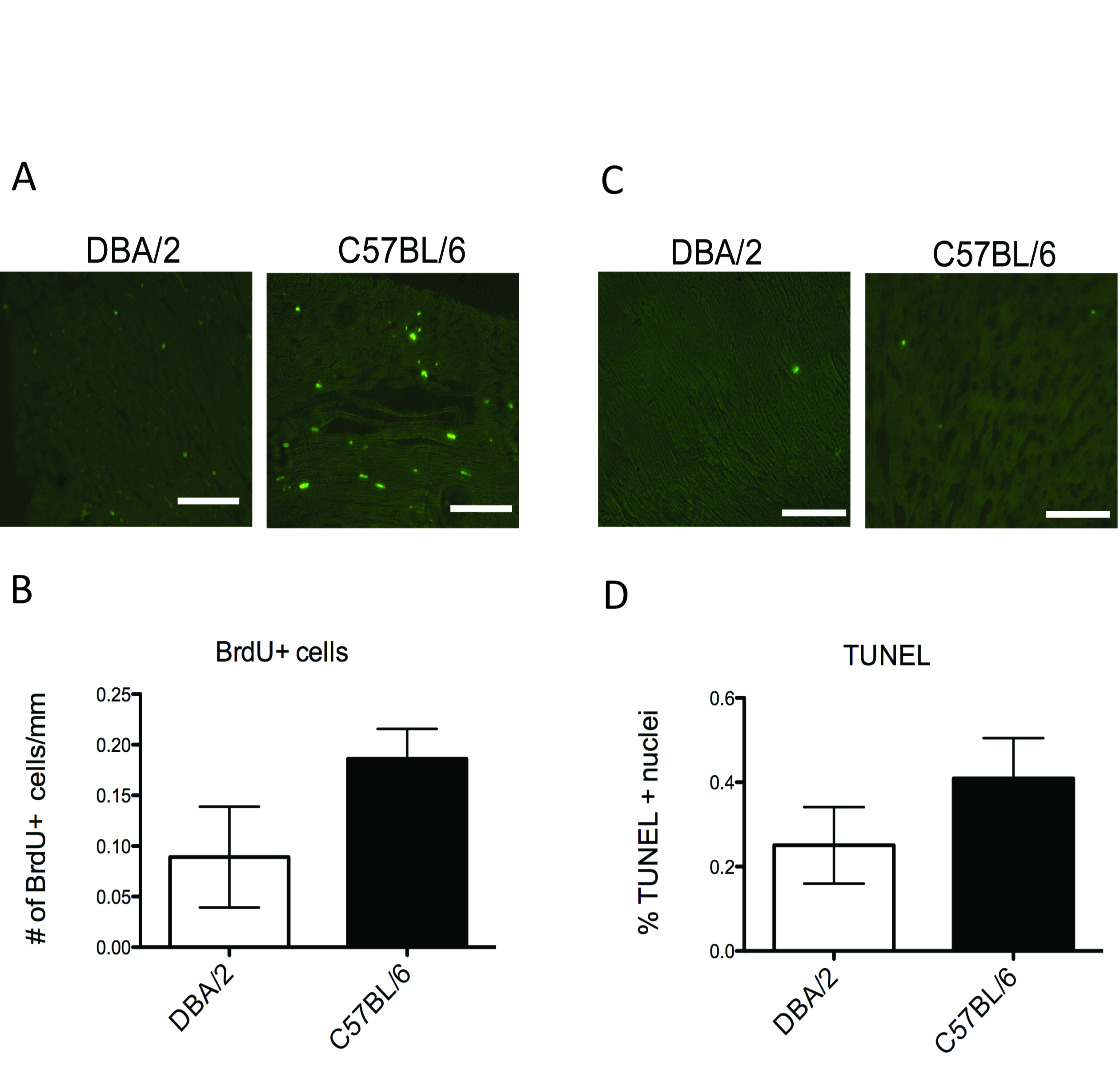

Supplement: Figure S1 — A) Representative picture of mitotic activity manifested as BrdU staining. Scale bar represents 100µm. B) Representative picture of apoptotic activity assessed by TUNEL staining. Below: Dapi staining of same slices. Scale bar = 100µm. C) C57BL/6 mice have a higher although not significant number of mitotically active cells in the heart compared to DBA/2 mice, n = 4. D) C57BL/6 mice have a higher number of apoptotically active cells in the heart compared to DBA/2 mice, however not statistically significant, n = 5. (TIF) [file pone.0070512.s001.tif]

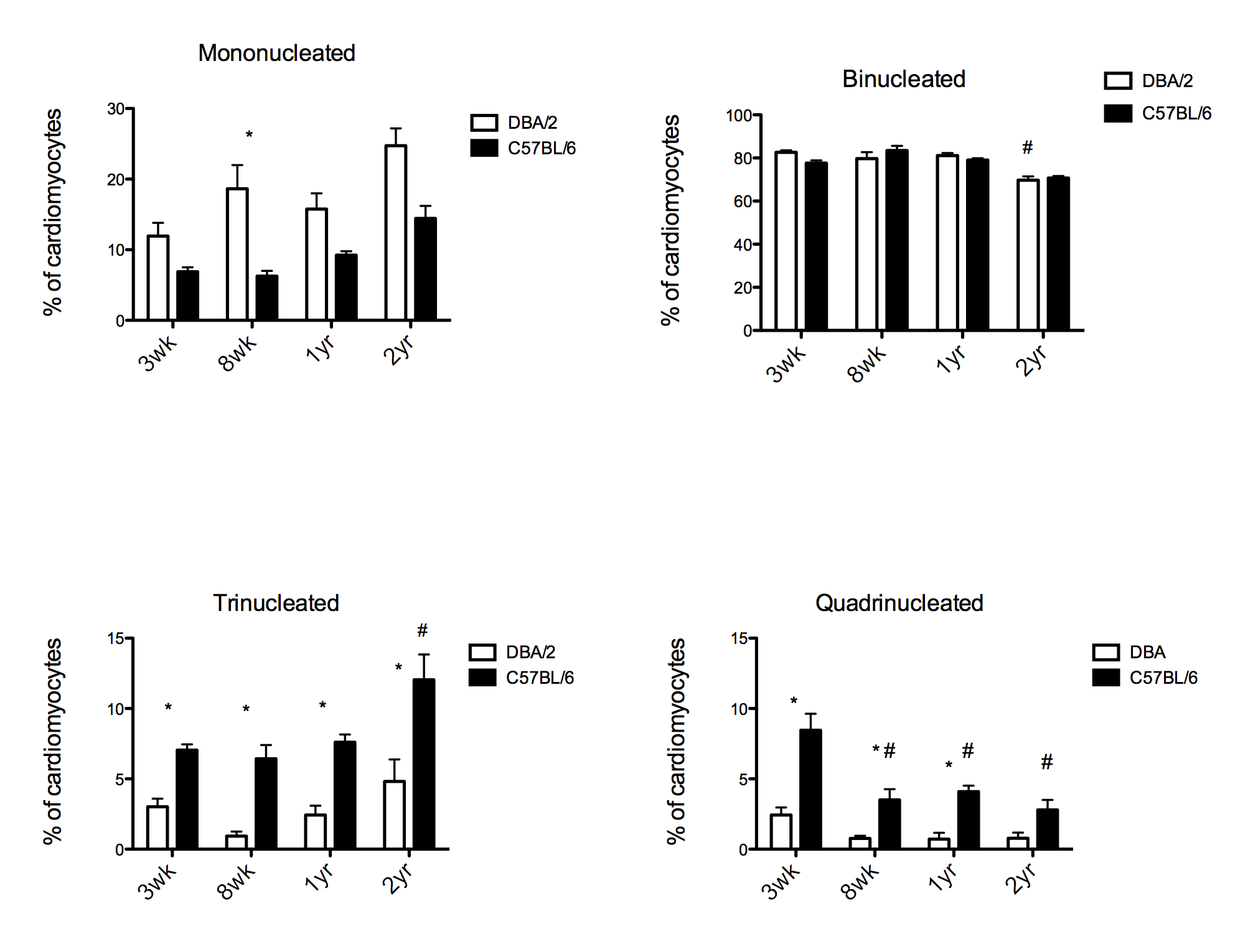

Supplement: Figure S2 — A) Mononucleated cardiomyocyte percentages remain higher in DBA/2 mice from age 3wks to 2yrs. B) Binucleated cardiomyocyte percentages are similar in both strains from age 3wks to 2yrs. And tri-nucleated C) and quadri-nucleated D) cardiomyocytes percentages are higher in C57BL/6 mice from age 3wks to 2yrs. (TIF) [file pone.0070512.s002.tif]
